# Effects of acute psychosocial stress on source level EEG power and functional connectivity measures

**DOI:** 10.1038/s41598-023-35808-y

**Published:** 2023-05-31

**Authors:** Gert Vanhollebeke, Mitchel Kappen, Rudi De Raedt, Chris Baeken, Pieter van Mierlo, Marie-Anne Vanderhasselt

**Affiliations:** 1 Department of Head and Skin, Ghent Experimental Psychiatry (GHEP) Lab, University Hospital Ghent, Ghent University, C. Heymanslaan 10, Entrance 12 – Floor 13, 9000 Ghent, Belgium; 2 Medical Image and Signal Processing Group (MEDISIP), Department of Electronics and Information Systems, Ghent University, Ghent, Belgium; 3 Department of Experimental Clinical and Health Psychology, Ghent University, Ghent, Belgium; 4 Department of Psychiatry, University Hospital (UZBrussel), Brussels, Belgium

**Keywords:** Neuroscience, Psychology, Neurology

## Abstract

The usage of EEG to uncover the influence of psychosocial stressors (PSSs) on neural activity has gained significant attention throughout recent years, but the results are often troubled by confounding stressor types. To investigate the effect of PSSs alone on neural activity, we employed a paradigm where participants are exposed to negative peer comparison as PSS, while other possible stressors are kept constant, and compared this with a condition where participants received neutral feedback. We analyzed commonly used sensor level EEG indices (frontal theta, alpha, and beta power) and further investigated whether source level power and functional connectivity (i.e., the temporal dependence between spatially seperated brain regions) measures, which have to our knowledge not yet been used, are more sensitive to PSSs than sensor level-derived EEG measures. Our results show that on sensor level, no significant frontal power changes are present (all *p*’s > 0.16), indicating that sensor level frontal power measures are not sensitive enough to be affected by only PSSs. On source level, we find increased alpha power (indicative of decreased cortical activity) in the left- and right precuneus and right posterior cingulate cortex (all *p*’s < 0.03) and increased functional connectivity between the left- and right precuneus (*p* < 0.001), indicating that acute, trial based PSSs lead to decreased precuneus/PCC activity, and possibly indicates a temporary disruption in the self-referential neural processes of an individual.

## Introduction

Stress can be defined as the mental and physical reaction to personal or environmental stimuli that are deemed threatening to an individual^1^. Research has consistently shown that, when endured for a prolonged time, stress negatively impacts both the onset and progression of a variety of illnesses such as coronary heart disease, depression, and anxiety disorder^2–5^.

Given this repeatedly reported link between stress and disease, a significant amount of research has been dedicated towards better understanding how stress affects individuals, and which stimuli lead to a stress response^6,7^. *Psychosocial* stress has been identified as one of the most important forms of stress throughout an individual’s life given its strong link with the development of psychopathology^8^. Psychosocial stress, present in either unpredictable or uncontrollable social situations which are deemed unpleasant or threatening^9^, has obtained its prominent position due to the abundance of social interactions throughout daily life^8,10–12^.

The role of the brain in the perception of stimuli as stressful and its reaction to stressors as the controlling agent of the following stress response has been a central focus of psychosocial stress research^13–15^. Initially, brain activity related to psychosocial stress has been studied mainly with functional magnetic resonance imaging (fMRI), and multiple brain regions have been identified that are involved in the psychosocial stress response. Cortical regions commonly found are the anterior insula (often coactive with parts of the inferior frontal gyrus such as the pars triangularis and pars opercularis), the anterior and posterior cingulate gyrus (ACC, PCC), the precuneus (often coactive with the PCC), and the orbitofrontal cortex (for various systematic reviews and meta-analyses, see^13,16–20^). Subcortical regions such as the (para)hippocampus, thalamus, lentiform nucleus, caudate nucleus, putamen, and amygdala are also consistently reported to be involved^13,16,19^. Aside from fMRI, electroencephalography (EEG) has been employed increasingly throughout recent years for the investigation of psychosocial stress-related brain activity. Our recent systematic review identified a total of 13 EEG measures that have been employed in psychosocial stress research^9^. Most commonly employed is frontal alpha asymmetry (with conflicting results between studies), alpha power (which decreases significantly due to psychosocial stressors), and beta power (which generally increases, although not significantly in our meta-analysis). Less commonly utilized measures are other power measures such as delta, theta, and sigma power, power ratios (the combination of power values from spatially distinct electrodes or from different frequency bands) and functional connectivity (FC, the study of temporal dependence between spatially distinct neural events^21^) measures^9^. EEG is also increasingly used for the detection of mental stress with machine learning, again showing the rise of this neuroimaging technique in stress research^22^.

A variety of neuroimaging-compatible paradigms have been developed for the investigation of psychosocial stress. Although all paradigms employ a psychosocial stressor (e.g., negative feedback and peer comparison in the Montreal Imaging Stress Task (MIST^23^), social exclusion in the Cyberball paradigm^24^, or social-evaluative threat in the Trier Social Stress Test (TSST^25^)), these psychosocial stressors are often accompanied by other stressors such as cognitive stressors (e.g., imposed time limits or task demands). This co-occurrence of stressor types makes it difficult to directly link the measured neural activity to the unique social aspect of the employed paradigm. Research has shown that different psychosocial stress paradigms evoke different neuronal responses from individuals^16^, and a significant amount of research is being conducted for the development of EEG-based systems for the detection of psychosocial stress^22^, so any ambiguity in neural activity changes due to co-occurring stressor types needs further clarification.

In a recent article, Ehrhardt and colleagues (2021) have explicitly investigated the contribution of various individual stressor types (cognitive effort, time pressure, and social-evaluative threat) to changes in alpha and beta band power of frontal electrodes (F7, F3, Fz, Fpz, F4 and F8). The sobering results from their analysis have shown that the employed psychosocial stressor (social-evaluative threat, the fear of being judged negatively^26^) does not significantly alter power in either the alpha or beta band, indicating that results attributed to the social component of a stress paradigm instead seem to reflect changes in cognitive processing^27^.

Although this implication is highly significant for the research field, psychosocial stress may be detectable by other EEG measures than frontal alpha or beta power. Sensor level-derived EEG measures are known to be affected by volume conduction, understood as the spreading of electrical signals from a single brain source throughout the head^28,29^. Psychosocial stressor-induced neural changes might therefore not be sufficiently registered by sensor level-derived EEG measures or can be overpowered by other spontaneous brain activity^30^. The usage of EEG source imaging, which projects the signals measured at the electrodes back to the neural sources within the brain^31^, and the corresponding source space is therefore of special interest. Aside from source level power measures, functional connectivity measures might also capture changes induced by psychosocial stressors and thus give more insight into the neuronal psychosocial stress response.

To investigate whether purely psychosocial stressors affect source level-derived EEG indices, we developed a paradigm where participants were exposed to a psychosocial stressor while keeping co-occurring stressors such as time pressure or task demands constant between both conditions. Participants were instructed to solve Raven’s matrices of different levels of difficulty^32^. After each matrix, participants received (comparative) feedback which was manipulated to induce psychosocial stress. In the control condition, participants received neutral feedback (i.e., the participant performs on par with other individuals), and in another condition, the negative condition, negative feedback (i.e., the participant performs (progressively) worse than other individuals). Time limits were kept equal between both conditions and to further eliminate possible interferences of the task itself, and only data collected during the feedback exposure were analyzed. To evaluate whether the applied stressor was successful in eliciting a stress response, electrocardiography (ECG) data and state questionnaires, self-assessment manikins (SAM^33^), were also collected throughout the study. The SAM contains two scales: arousal (degree of activation due to the stimuli, from low to high) and valence (experienced emotional reaction to the stimuli, from negative to positive).

The research questions of the current study are threefold. Firstly, we investigated whether the psychosocial stressor elicits a physiological and mental response from the participants. We hypothesized that in the ECG signal, similarly to other psychosocial stressors, we would find an increase in sympathetic reactivity, identified by an increased heart rate acceleration, during the negative-, compared to the control condition^34–36^. We further hypothesized that in the SAM, in line with prior research, an increase in the arousal scale and a decrease in the valence scale would be found^37^. Secondly, we tried to reproduce the results found by Ehrhardt and colleagues (2021) and therefore computed frontal theta, alpha, and beta power at the sensor level, and compared the negative to the control condition. We hypothesized that similar to those results, no changes in these commonly used EEG measures due to a psychosocial stressor alone would be found. Finally, we investigated whether the purely psychosocial aspect of a stressor would be effective enough to affect source level-derived EEG measures. Therefore, we investigated the cortical regions commonly found in fMRI research (i.e., the anterior insula, ACC, PCC, precuneus, and orbitofrontal cortex; see above) and computed both their band power (theta, alpha, and beta) and the functional connectivity between them. Functional connectivity was estimated using amplitude envelope correlation (AEC), a robust connectivity measure^38,39^. Given previous fMRI research, we hypothesized an increase in beta power in the anterior insula and an increase in alpha power in the precuneus and PCC^16^. We had no specific directional hypotheses regarding the functional connectivity estimates.

## Results

Given that this article is part of a larger project, the SAM and ECG analysis (sections  "ECG results" and "SAM results") have also been described in another article^40^. We refer the interested reader to the aforementioned article by Kappen and colleagues (2022) for further information.

### ECG results

A Generalized Linear Mixed Model (GLMM) with gama distribution (R formula = *IBI-difference* ~ *Condition* + *(1|Participant_ID)*) revealed a significant interaction effect for *IBI-difference x Condition*. During the negative feedback, IBI_2_ to IBI_7_ (with IBI_0_ being the one closest to the feedback onset) were significantly lower than during the control feedback (*p*’s <  = 0.001), thus indicating that negative feedback resulted in an increased heart rate acceleration, confirming our hypothesis that negative feedback would result in an increase in sympathetic reactivity.

### SAM results

The GLMMs with gamma distributions (one for each axis of the SAM; R formula = *Scale_score* ~ *Condition* + *(1|Participant_ID)*) revealed a significant C*ondition* effect for both valence and arousal. Valence decreased significantly during the negative condition compared to the control condition (*p* < 0.001), confirming our hypothesis^37^. Arousal also decreased significantly between the control condition and negative condition (*p* = 0.034), contradicting our hypothesis^37^.

### EEG results

A summary of all EEG results (regardless of significance) can be found in the supplementary materials (link: https://osf.io/xjstp). In the following sections, only significant results will be described.

#### Sensor level

The frequentist statistical analysis from GLMMs with gamma distributions (R formula = *PowerValue* ~ *Condition* + *(1|Participant)* indicated that no significant changes for the sensor level analyses (frontal theta, alpha and beta power) were found (*p’*s > 0.16). The subsequent Bayesian statistical analysis provided moderate evidence in favor of the null hypothesis for theta power (BF01 = 6.40) and beta power (BF01 = 8.79). For alpha power, the Bayesian analysis provided anecdotal evidence in favor of the null hypothesis (BF01 = 1.24).

#### Source level

Five source level results are significant, all significant results are from GLMMs with gamma distributions (R formula = *PowerValue* ~ *Condition* + *(1|Participant)* for relative power measures ; R formula = *FCValue* ~ *Condition* + *(1|Participant)* for functional connectivity (FC) measures). The relative alpha power of the right posterior cingulate cortex (*ß* = 0.018; *SE* = 0.0042; *t* = 4.202; *p* < 0.001; *standardized effect size (SES)* = 0.1 with *95% confidence interval (CI)* = 0.05; 0.15), left precuneus (*ß* = 0.013; *SE* = 0.004; *t* = 3.124; *p* = 0.03; *SES* = 0.07; *CI* = 0.03;0.12) and right precuneus (*ß* = 0.02; *SE* = 0.004; *t* = 4.385; *p* < 0.001; *SES* = 0.11, *CI* = 0.06; 0.16) all increase significantly in the negative- compared to the control condition, confirming our hypothesis. The functional connection in the alpha frequency range between the left- and right precuneus (*ß* = 0.017; *SE* = 0.002; *t* = 8.188; *p* < 0.001; *SES* = 0.08; CI = 0.06; 0.1)) also increased in the negative condition. One further functional connection (between the right PCC and right precuneus in the beta frequency range) remained significant after multiple comparison correction, but the model failed to converge due to the minimal difference between conditions, and thus will not be discussed further. All other analyses revealed no significant differences between conditions (*p*’s > 0.15), contradicting our other hypotheses regarding an increase in beta power in the anterior insulae^16^. For all models, sex did not improve the models significantly (all *p*’s > 0.12).

## Discussion and conclusion

Previous research investigating the neural signature of the psychosocial stress response through means of EEG has identified changes in several EEG indices, most notably band power^9^. A recent article by Ehrhardt and colleagues (2021), however, has shown that two of the most commonly investigated indices, frontal alpha, and beta power, do not seem sensitive enough to change significantly by psychosocial stressors alone. Previously reported alfa and beta band power changes might therefore not reflect the influence of psychosocial stressors themselves, but rather the influence of co-occurring stressors such as time pressure or cognitive processes related to the task at hand. While this insight is puzzling and demands reflection within the research field, it is possible that other EEG indices, such as source level-derived power and functional connectivity measures, do change significantly due to psychosocial stressors alone. To investigate this, we exposed a large sample of healthy adults to a psychosocial stressor using a within-subjects design by providing manipulated feedback. Participants were shown either a personal performance on par with a comparison group (*control* condition) or a worse personal performance compared to a group of high-achieving peers (*negative* condition). We kept other stressors such as time limits or cognitive tasks constant in both conditions and to further exclude cognitive processing-related oscillatory interferences, we only analyzed the EEG data collected during feedback exposure. Aside from EEG data, we also collected ECG data to investigate whether psychosocial stressors lead to a short-term physiological reaction and a state questionnaire, the self-assessment manikin, to probe the mental state of the participants.

Analysis of the IBIs from the ECG data during the feedback revealed that during the negative condition, the IBI-difference from IBI_2_ to IBI_7_ was significantly shorter than during the control condition, indicating a higher heart rate acceleration during the negative condition^40^. Heart rate acceleration is a sign of sympathetic nervous (re)activity, which is known to be increased due to stress^35,36,41^. The higher heart rate acceleration during the negative condition, therefore, indicates a higher short-term sympathetic reactivity, indicating the effect of the psychosocial stressor. Results of the SAM showed a significant decrease in both valence and arousal. The decrease in valence aligns with our hypothesis and indicates the effect of the negative feedback on the mood of the participants. The decrease in arousal does not align with our hypothesis, and might reflect a possible order effect due to the control condition being before the negative condition or an unclear translation of the word *arousal* to Dutch (which has the same translation of the English word *excitement*). Given the increased heart rate acceleration and decrease in valence in the negative condition however, we conclude that the psychosocial stress induction was successfull.

Analysis of the EEG data shows that on the sensor level, frequentist statistical analysis revealed no significant changes in either theta, alpha, or beta power of frontal electrodes between conditions. These results reaffirm the results of Ehrhardt and colleagues^27^ and show that, when employing frequentist statistics, psychosocial stressors alone are not capable of inducing significant changes in these commonly employed sensor level EEG measures. The subsequent Bayesian analyses further provided moderate evidence in favor of the null hypothesis (i.e., psychosocial stressors do not lead to significant changes in sensor level band power of frontal electrodes) for theta and beta power. For theta power, the BF01 was 6.4, suggesting that our results are 6.4 times more likely to be observed under the null hypothesis. Our recent systematic review found contradicting results regarding theta power, which might be explained by the fact that this EEG measure is not sensitive enough to detect psychosocial stress related neuronal changes, as our results suggest^9^. Similarly, the BF01 for beta power was 8.79, suggesting that the null hypothesis is 8.79 more likely given our results. This result is also in line with our recent meta-analysis, which revealed a non-significant effect size for beta power across studies^9^. Contrary to the Bayes factors for theta and beta power, only anecdotal evidence in favor of the null hypothesis for alpha power was found (BF01 being 1.24). Our meta-analysis for alpha power identified a significant effect size, and alpha power was found to be the best feature for mental stress detection in another recent review^9,22^. Although our results regarding alpha power at frontal electrodes align with those of Ehrhardt and colleagues^27^, no strong evidence was found for the null hypothesis either. Future studies should therefore further examine the exact influence of psychosocial stressors on frontal alpha power changes. While the weight of this conclusion cannot be ignored, it should be noted that in both the article of Ehrhardt and colleagues (2021) and the current article, subtle psychosocial stressors are employed. Ehrhardt and colleagues (2021) used a video camera and the announcement of the analysis of performance and behavior while in the current article manipulated feedback was employed as a psychosocial stressor. It is possible that more potent psychosocial stressors, such as direct exposure to an unfriendly panel of experts in the TSST^25^, are capable of inducing sensor level EEG changes (for an overview of articles that use the TSST with EEG, see the supplementary materials of^9^). Further is it also possible that band power changes in other electrodes, aside from those investigated in the current article, are sensitive to psychosocial stressor induced neural changes. An exploratory whole-brain, sensor-level analysis for theta, alpha, and beta power was conducted (link: https://osf.io/epk8c) that indicated no significant changes in the theta and beta range (theta power results: https://osf.io/zepu4; beta power results: https://osf.io/dq27w), but showed that alpha power changed significantly between the neutral and negative feedback condition for several parietal electrodes (link: https://osf.io/pkejw). These significant changes might reflect the observed effect in the precuneus and PCC and demonstrate the value of assessing psychosocial stress at electrodes outside of the commonly investigated frontal electrodes^9^. Regardless, technical problems in sensor level analyses related to volume conduction, whereby activity of (mainly) occipital neural generators are picked up by frontal electrodes, should be considered and sensor level power measures therefore, need to be interpreted with severe caution^29^.

In the source space, in contrast to the non-significant results of the commonly employed sensor level EEG indices, a significant increase in alpha power in the right precuneus and posterior cingulate cortex (PCC), as well as the left precuneus, are found in the negative, compared to the control condition. Furthermore, an increase in functional connectivity between the left and right precuneus, computed using amplitude envelope correlation, was observed. The significant changes found in source space all point to a single direction: an increase of activity and connectivity in the alpha band of the precuneus/PCC complex. Oscillations in the alpha frequency range are assumed to reflect an inhibitory coordination system within the brain^42,43^ and increases in alpha power are therefore expected to reflect decreases in cortical activity^44^. Our results thus implicate that a short-term psychosocial stressor leads to an acute decrease in cortical activity of the precuneus/PCC cluster. This decrease in precuneus/PCC activity aligns with a recent meta-analysis of fMRI studies studying psychosocial stress, which also found a decrease in (BOLD) activity in the precuneus and PCC^16^. Interestingly, studies investigating trial-based manipulation-free social comparisons also report changes in precuneus and PCC activity^45,46^. These studies, however, sometimes find increased precuneus/PCC activity, an incongruence also identified in another review of fMRI studies employing a variety of stressors^19^. This incongruence mainly highlights the complex interactions within the brain and indicates the necessity for further investigation. Finally, precuneus/PCC activity is also found in an EEG study investigating social comparisons^47^. This study, which employed event-related potentials (ERPs) to investigate social comparisons, identified the (pre)cuneus as the generator of an ERP (early negativity) when participants felt shameful in a social context, linking the cluster again to negative social comparison^47^. Taken together, our results show that uncontrollable negative peer comparison leads to decreased activity of the precuneus/PCC complex.

The precuneus/PCC complex is a key region of the default mode network (DMN), a network active when no external tasks are presented to an individual which has been linked with self-reflective, internally directed thoughts^48^. Several studies have also identified increased activity in the precunes/PCC complex during tasks related to self-reflection^49–51^. These activations can be explained by the integrative model of the PCC from Cavanna and colleagues, which poses that PCC activity increases when thoughts are more internally focussed^52^. Consequently, decreased activity of the precuneus and PCC have also been linked with tasks that are less self oriented when compared to more self-oriented tasks^50^. Activity changes in the precuneus and PCC have further been reported during emotion regulation. In a recent article of Guendelman and colleagues, it was shown that increased activity of the precuneus/PCC (contrary to our findings of decreased activity) was associated with both lower self-reported stress as well as decreased autonomic sympathetic activation during emotion regulation^53^. Increased precuneus activity has also been reported in women with higher self-compassion, and was further linked with decreased levels of perceived stress during the viewing high arousal negative valence pictures^54^. Disrupted precuneus/PCC activity is further identified in several stress-related psychiatric disorders, such as post-traumatic stress disorder^55^, social anxiety disorder^56^, and depression^57^. Taken together, our results of decreased precuneus/PCC activity might thus imply a short-term attentional shift from internal towards external focus for the regulation of an acute external threat (i.e., a psychosocial stressor)^52,58,59^.

While our results are promising and show that psychosocial stressors do lead to significant changes in EEG indices, it should be noted that these observed changes are small. The likely reason for the small effect sizes is the subtlety of the employed stressor and inherently limited sensitivity of EEG as a neuroimaging technique^30^. In conclusion, in this article, we have shown that source level-derived EEG indices are, contrary to the commonly utilized sensor level-derived indices alpha and beta power of frontal electrodes, sensitive enough to investigate neuronal changes due to purely psychosocial stressors. The modest effect sizes hint at the limited capability of EEG to capture subtle mental changes in an individual. We therefore advise other researchers in the field to (1) use large participant groups; (2) employ within-subject designs and (3) use potent psychosocial stressors such as the TSST to further investigate the effects of pure psychosocial stressors on neural activity, as measured by EEG.

## Materials and methods

### Participants

A convenience sample of eighty-three healthy, Dutch-speaking individuals was recruited from the general population through internet postings on social media. All participants were right-handed, had no personal or familial history of epilepsy, have not had any neurosurgical procedure throughout their life, did not have any psychiatric-, neurological-, substance abuse-, heart-, respiratory-, or eye disorder in their life, had no metal or magnetic objects in their body or brain, were not using any psychoactive medication and had no skin conditions at the level of the head. All participants refrained from caffeine and nicotine in the two hours leading up to the experiment. Data was collected between 10 a.m. and 5 p.m. Data from 10 participants was not used (two participants had incomplete data, and eight participants were excluded due to insufficient EEG data quality based on visual inspection or remaining epoch amount after artifact rejection), resulting in a final dataset of 73 participants (47 females, M_age_ = 22.8, SD_age_ = 5.3, Age range = 18–47 years). The experiment was conducted in accordance with the Declaration of Helsinki and was approved by the Medical Ethical Committee of the Ghent University Hospital (registration number: B670201940636). Participants received €30 for their participation.

### Experimental procedure

#### Study paradigm

Before in-person data collection, participants gave their informed consent and filled in trait questionnaires (this study is part of a larger project, and these trait questionnaires are not further discussed in this article) through the online platform Limesurvey^60^. In-person data was collected in a dedicated room at the Department of Neurology at the Ghent University hospital. Upon arrival, participants gave their written consent again (on paper), after which the EEG and ECG electrodes were applied (total duration between 20 and 45 min). After electrode placement, participants were seated in a chair in front of a computer monitor (Dell E2216H) at a distance of around 60 cm and were told to remain seated and move as little as possible to reduce the presence of motion artifacts in the data. Instructions and tasks for the experiment were given using E-Prime 2.0 (Psychology Software Tools, Pittsburgh, PA) and the SAMs were collected using a custom app on a tablet (Huawei MediaPad M5).

**Figure 1:**
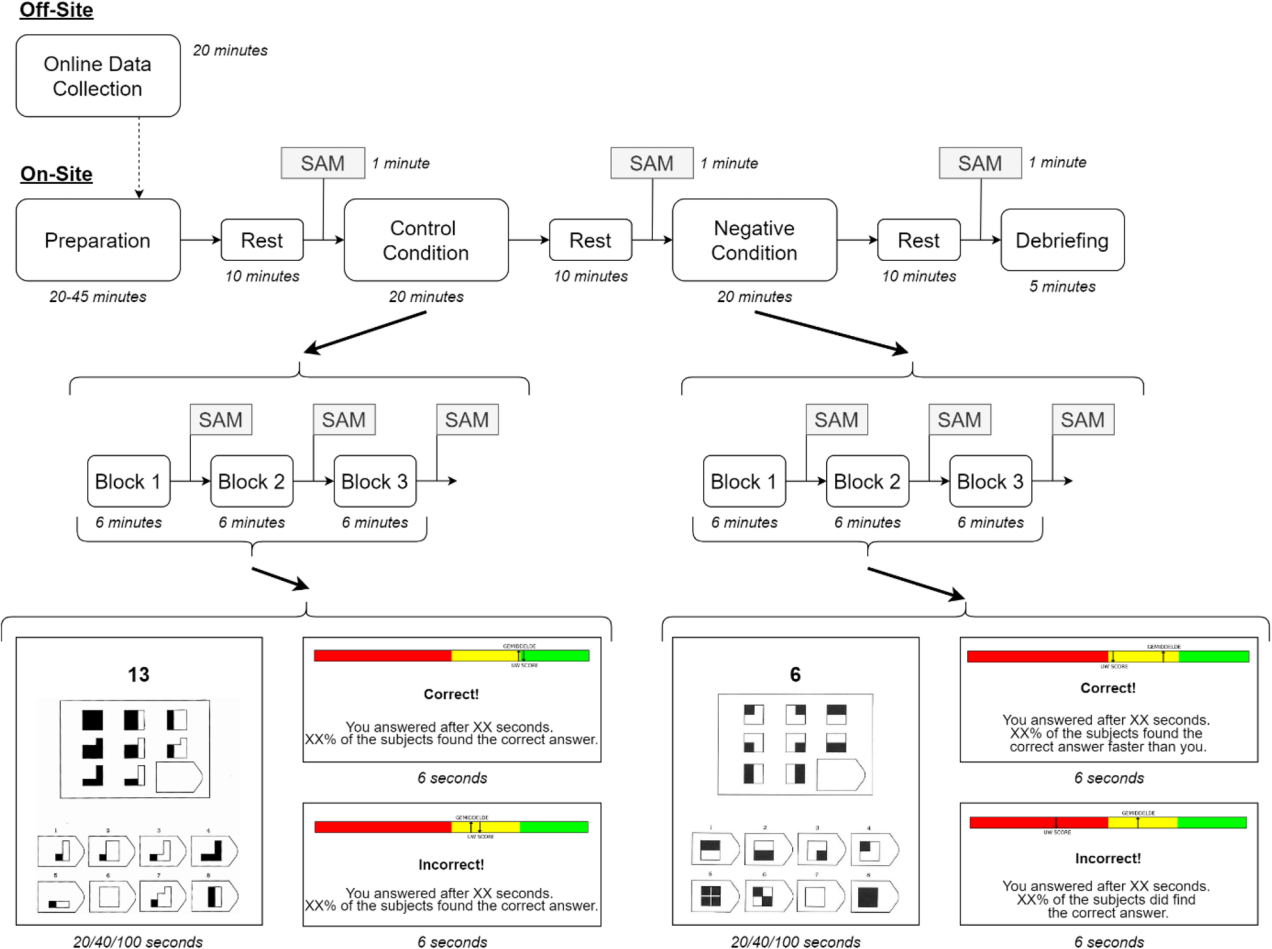
Visualization of the study paradigm. *Off-Site*: start of the study where a participant reads and signs the informed consent and afterwards completes the trait questionnaires. *On-Site*: part of the study where the participant comes to the University Hospital. *SAM*: moment when a self-assessment manikin questionnaire is recorded. *Preparation*: participant signs the informed consent again, EEG and other electrodes are applied. *Rest*: Resting-State, eyes closed EEG recording. *Control Condition*: participant performs the task, with neutral feedback. *Negative Condition*: participants performs the task, but receives negative feedback, regardless of their performance. *Debriefing*: end of the experiment. Participants are told about the goal of the study, are told that the feedback in the negative condition was not real, and are thanked for their participation. *Block 1/2/3*: block of a condition. Each block lasts either 6 min, or ends when a participant has solved all 11 Raven’s Matrices assigned (randomly) to the specific block.

After the introduction and electrode placement, participants rested for 10 min with closed eyes (habituation), after which they filled in a SAM questionnaire (see section "Self-assessment manikin"). Following the initial resting period, the control condition was presented. In the control condition, participants solved a series of Raven’s matrices (see section "Trial and feedback") in three blocks, with each block either lasting six minutes or ending when the participant solved all 11 matrices assigned to the block. After each block, participants filled in a SAM. After the control condition, participants rested again for 10 min (followed by a SAM) after which the negative condition was presented. The negative condition was identical to the control condition, aside from a manipulation during the feedback (see section "Trial and feedback"). After the negative condition, participants rested for a third time for 10 min (followed by a final SAM), after which they were debriefed about the goal of the study, and payment information was collected. The study paradigm is shown in Fig. 1.

#### Trial and feedback

In both the control and negative condition, participants were instructed to solve Raven’s matrices. Raven’s matrices are a visual exercise where eight figures are presented in a 3 × 3 raster with an empty space in the lower right part of the raster (see Fig. 1, bottom for an example). The goal of the exercise is to select the ninth figure (from 8 possibilities) which completes the raster by identifying the pattern shared by the eight initially shown figures^32^. Shown above the Raven’s matrix was a countdown timer, which showed the time left to solve the Raven’s matrix in seconds. Three levels of difficulty were defined for the Raven’s matrices and the allowed time to solve each problem depended on the difficulty of the individual matrix (20 s for easy, 45 s for medium, and 100 s for difficult; see OSF (link: https://osf.io/py63g) for further information regarding the Raven’s matrices). Participants solved the matrices by pressing a number between 1 and 8 with their right hand on the keyboard Numpad, corresponding to one of the eight possible solutions. When the Raven’s matrix ended (either through a response of the participant, or a time-out) a feedback screen consisting of three components was shown for six seconds. The first component (*top*) was a three-colored (red, yellow, green) comparison bar containing two arrows indicating 1) the individual and 2) the average performance of a comparison group; the second component (*center*) was a single word indicating the evaluation of the participant’s response (“correct!”, “incorrect!” or “time-out!”); the third component (*bottom*) was a short text showing the participant how long it took him/her to solve the matrix, and a comparison to the aforementioned average performance (see Fig. 1, bottom).

#### Cover story and manipulation

To induce psychosocial stress, participants were told that the study investigated possible EEG indices that might be indicative of future (either academic or professional) success in life. Participants were told that to investigate this, they would solve a well-known IQ test (i.e., Raven’s matrices) while EEG data was collected. Participants were further told that to assess future success, their performance would be compared with two groups. Firstly, they would be compared with a control group of average individuals (i.e., the control condition) and afterward, they would be compared to individuals which achieved significant academic or professional success in their life (i.e., the negative condition). Finally, participants were told that their performance was calculated based on both the correctness of their answers and the time it took to solve each trial.

The manipulation happened in the feedback that the participants received. During the control condition, regardless of the performance of the participant, the feedback showed that the individual performance was about equal to the average performance of the comparison group. This was shown in the comparison bar (top) and the short text (bottom), which declared what percentage of the subjects in the comparison group found the correct answer. During the negative condition, in each block participants’ performance started roughly on par with the comparison group, but became progressively worse while the participant progressed through the block, regardless of the true performance of the participant (see supplemental Fig. 2; link: https://osf.io/wpukf). The progression from being on par to performing below the average was chosen to increase the believability of the feedback. This was shown in the comparison bar, which indicated that the personal score decreased while the average score remained similar throughout the exercises. The short sentence at the bottom also changed slightly, now indicating what percentage of people found the answer quicker than the participant when they answered correctly, or which amount of participants did find the correct answer when the participant answered incorrectly or did not give an answer in time (see Fig. 1, bottom).

#### Self-assessment manikin

To assess the mental state of the participants throughout the experiment, the self-assessment manikin (SAM), a non-verbal questionnaire that assesses the state affective reaction of an individual, was conducted^33,61^. The SAM consists of three rows, each containing 9 pictures, indicating various levels of arousal and valence, and the participants selected a picture in each row that best corresponded to their emotional state at that moment. The picture scale corresponds to a Likert scale (range 1–9). The SAM was chosen, aside from its simple design and easy interpretation, to make it less likely that participants became aware of the goal of the study (i.e., repeated questions related to “stress” or “negative feelings” might make participants suspicious). The pictures as well as the corresponding instructions can be found on OSF (picture link: https://osf.io/6sy5t; instruction link: https://osf.io/se7qj).

### ECG analysis

To assess whether negative feedback elicited a physiological reaction, the ECG data were analyzed during the feedback segments. Event-related cardiac responses, computed using inter-beat intervals (IBIs) which indicate the time between individual heartbeats, were therefore analyzed^62,63^. To analyze the feedback moments, the R-peak closest to the onset of the feedback was selected and the IBI compared to the previous R-peak was computed and defined as IBI_0_. From IBI_0_, the three preceding IBIs (IBI_-3_, IBI_-2_, IBI_-1_) and eight subsequent IBIs (IBI_1_ until IBI_8_) were also computed (see supplementary materials for more information, link: https://osf.io/yvzr5). All IBIs were then re-referenced to IBI_-2_, thus obtaining IBI-difference scores (similar to previous research, see Gunther Moor et al.^62^; van der Veen et al.^63^). Positive/negative IBI-difference scores can be interpreted as a heart rate acceleration/deceleration (compared to the reference IBI; IBI_-2_).

### EEG equipment and analysis

#### EEG equipment

EEG data was collected at 57 standard locations according to the international 10–10 system using a 64-channel, Ag/AgCl electrode Waveguard cap (ANT Neuro, the Netherlands) combined with a MICROMED SD LTM 64 EEG amplifier (Micromed S.p.A., Mogliano, Italy). Cz was used for online referencing while AFz was used as ground. Given the limited recording channels of the amplifier, four channels (PO7, PO8, O1, O2) were omitted from EEG recording for physiological data recording (electrocardiography (ECG) and electrodermal activity (EDA))). Electrode impedances were kept below 20 kΩ during data acquisition and data was collected at a sampling rate of 512 Hz. Data was filtered online using a high-pass filter at 0.008 Hz.

#### Preprocessing

EEG data were preprocessed using BrainVision Analyzer (Version 2.1., Brain Products GmbH, Gilching, Germany). Before preprocessing, the complete control and negative condition segments were extracted from the continuous EEG recording. The following preprocessing steps were performed for both EEG segments identically. Firstly, irrelevant channels for EEG preprocessing (i.e., ECG and EDA channels) were removed. Secondly, all data were filtered (50 Hz (Notch Filter), 1–40 Hz (IIR bandpass filter, 48 decibels/octave)). Thirdly, bad channels (channels with high amounts of electrical noise, identified by visual inspection) were interpolated (topographic spline interpolation, spline order = 4, maximal degree of Legendre polynomials = 10, lambda = 1e-5). Fourthly, Independent Component Analysis (ICA) with standard settings was performed and components representing eye movements, heart rhythm activity, or muscle movement were manually selected (based on their topography and time course) and removed. Afterward, remaining artifacts were detected based on three criteria: gradient (maximum allowed voltage step of 50 µV/ms), min–max (maximum allowed voltage range of 200 µV/200 ms), and low activity (minimum of 0.5 µV/100 ms). Artifacts detected by this method were tagged 200 ms before and after the identified artifact. Epochs of 6.2 s were created based on the feedback triggers (200 ms before until 6 s (feedback exposure duration) after the trigger) and epochs containing artifacts were removed. Finally, the EEG data were re-referenced to an average reference, and data was exported in EDF + format for further analysis. A visual representation of the preprocessing pipeline and the preprocessed data (not the raw data) can be found on OSF (link: https://osf.io/qxmgy).

#### Sensor level analysis

For the sensor level analysis (i.e., analysis of the time series measured by the electrodes) average power in the theta (4–8 Hz), alpha (8–13 Hz), and beta (13–30 Hz) frequency band of 6 frontal electrodes (F7, F3, Fz, FPz, F4, F8) was computed. Preprocessed EEG data were first converted from EDF + format to MATLAB .mat files for further usage (these files can be found on OSF; link: https://osf.io/tywxp). Relative power, meaning the average power in a defined frequency band divided by the total power of the considered spectrum (1–40 Hz), was computed. Power of the EEG signals was computed using Welch's spectral power density estimate (MATLAB function: *pwelch*) and a 1/f noise correction is employed with the correction exponent equal to one^30^. Relative power was computed for each of the aforementioned electrodes separately and the average of these values is calculated to obtain a mean power estimate of the frontal cortical regions. All sensor level analyses were performed using custom code in MATLAB and can be found on OSF (link: https://osf.io/tywxp).

#### Source level analysis

##### Source modeling

EEG source modeling was performed using the Brainstorm Toolbox^64^. The USCBrain atlas and corresponding T1 weighted MRI image were used for this processing step as no individual MRI images of the participants were available^65^. The T1 image is an average image from five different high-resolution MRI scans from a single right-handed female. The EEG electrodes were co-registered to the MRI image using LPA, RPA, FPz, and Oz as landmarks and the obtained coordinates were converted to the corresponding MNI coordinates. To construct the head model, the SimNIBS *mri2mesh* Finite Element Method (FEM, known for its high spatial resolution^66^) was employed, resulting in a 5-layer (scalp, skull, gray matter, white matter, and cerebrospinal fluid) FEM mesh of 642,359 vertices^67,68^. To solve the forward problem and obtain the leadfield matrix, evenly spaced (5 mm) dipoles were defined in the gray matter (15,269 in total), and the placement of the electrodes was finalized by visual inspection using the aforementioned landmarks and projecting the electrodes directly on the scalp. The forward model and corresponding leadfield matrix were obtained using the DUNEURO toolbox within Brainstorm^69^. Isotropic conductivities were used (scalp = 0.43, skull = 0.008, GM = 0.33, WM = 0.14, CSF = 1.79) and the default options were used for the FEM solver type and source model. To solve the inverse problem, the orientation of the dipoles was constrained and set to be normal to the cortex and current density maps (unit Ampere-Meters) were obtained using the weighted minimum norm estimation method (wMNE, known for the limited spatial leakage^70,71^) with default options for depth weighting and regularization. Sensor noise was estimated using the diagonal of the noise covariance matrix. Finally, time series of all scouts of the USCBrain atlas (65 regions in each hemisphere, 130 in total) were obtained by taking the mean of all dipole values belonging to a scout. The obtained scout time series were extracted from the Brainstorm toolbox for subsequent analyses. The standard settings for the different steps can be found on OSF (link : https://osf.io/8t9rq).

##### Mapping of brain regions to atlas regions

**Figure 2:**
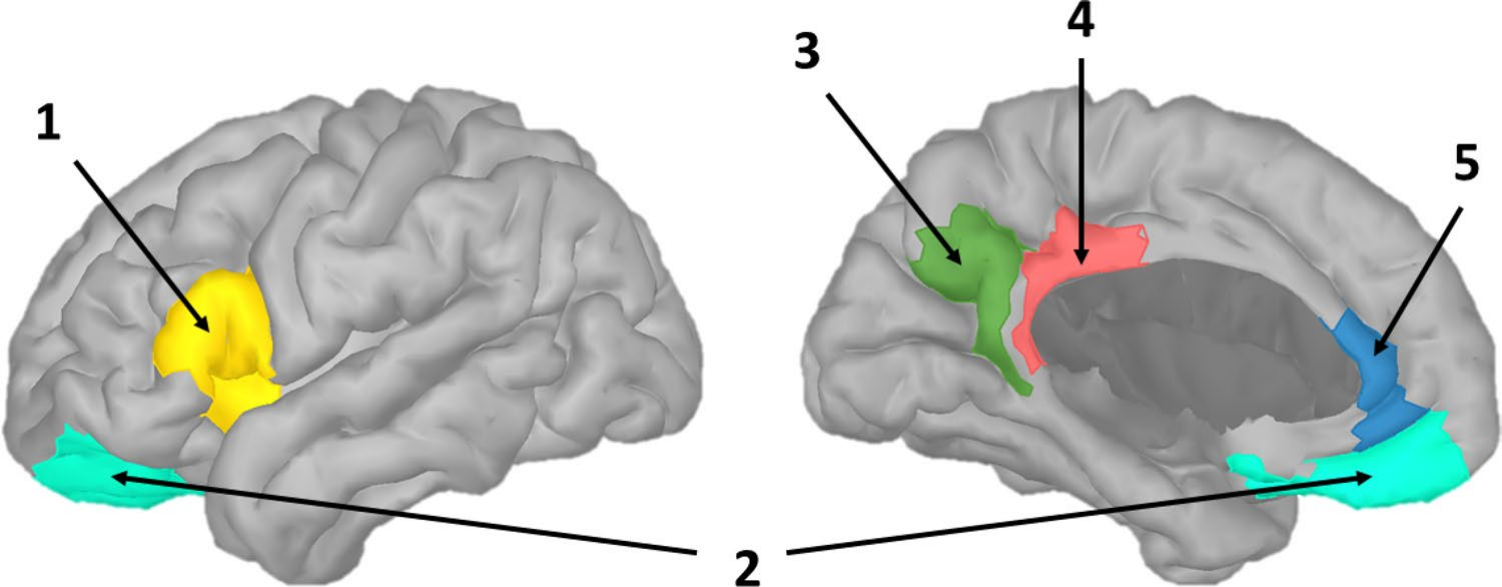
Visualization of the investigated brain ROIs. **(1)**: Anterior Insula; **(2)**: Orbitofrontal Cortex; **(3)**: Precuneus; **(4)**: Posterior Cingulate Cortex (PCC); **(5)**: Anterior Cingulate Cortex (ACC). *Note: Only the left hemisphere is shown in this figure, the contralateral ROIs are also included in the analyses.*

As indicated in the introduction, various brain regions of interest (ROI) have been identified in previous research^13,16–20^. Based on the literature, we selected 10 regions (five regions in each hemisphere) for further investigation: the *anterior insula* (combined with parts of the pars triangularis and opercularis), the *ACC*, the *PCC*, the *precuneus,* and the *orbitofrontal cortex*. Table 1 shows which regions (called scouts) of the USCBrain atlas have been selected for further analysis. Given the limited temporal resolution of EEG, multiple USCBrain scouts were combined for the anterior insula and orbitofrontal cortex to obtain brain regions that are within the spatial resolution possibilities of EEG. If multiple regions of the atlas were selected, a single time series was obtained by extracting the first principal component of the selected time series using principal component analysis. The selected brain ROIs are shown in Fig. 2.

**Table 1:** Conversion of brain ROIs to scouts of the USCBrain Atlas^65^.

Brain ROI	USCBrain Scouts (L)	USCBrain scouts (R)
Anterior insula	Insula—anterior L	Insula—anterior R
	Pars Opercularis—Inferior L	Pars Opercularis—Inferior R
	Pars Opercularis—Superior L	Pars Opercularis—Superior R
	Pars Triangularis—Middle L	Pars Triangularis—Middle R
	Pars Triangularis—Posterior L	Pars Triangularis—Posterior R
PCC	Cingulate Gyrus—Posterior L	Cingulate Gyrus—Posterior R
Precuneus	Precuneus—Inferior L	Precuneus—Inferior R
ACC	Cingulate Gyrus—Anterior L	Cingulate Gyrus—Anterior R
Orbitofrontal cortex	Anterior Orbito-frontal Gyrus L	Anterior Orbito-frontal Gyrus R
	Gyrus Rectus L	Gyrus Rectus R
	Middle Orbito-frontal Gyrus L	Middle Orbito-frontal Gyrus R

##### Power analysis

Power analysis on source level was conducted identically as on sensor level (see section "Sensor level analysis"). Relative power of the theta (4–8 Hz), alpha (8–13 Hz), and beta (13–30 Hz) frequency range was computed for the brain regions defined in Table 1. All source level power analyses were performed using custom code in MATLAB. The analysis scripts can be found on OSF (link: https://osf.io/tywxp).

##### Functional connectivity analysis

To assess functional connections between the brain ROIs (see Table 1), amplitude envelope correlation (AEC), a robust linear, undirected, and bivariate FC measure was used^38,39,72,73^. To calculate AEC, the time series of each brain region were band-passed so only the signals in a specific frequency range were considered (see Fig. 3.1). The band-passed signals were then pairwise orthogonalized (see Fig. 3.2.) using a stabilized Gram-Schmidt orthogonalization algorithm. Orthogonalization was performed to minimize the effect of spatial leakage due to the blurring effect of the weighted minimum norm estimation, which could lead to spurious functional connections^74^. Since this orthogonalization method is non-symmetric (i.e., GSO(Sig_1_, Sig_2_) does not equal GSO(Sig_2_, Sig_1_)), orthogonalization was performed twice and all steps described below were conducted on each pair of orthogonalized signals. The final FC value was obtained by averaging the two FC values. Next, the power envelope of the orthogonalized time series was obtained by applying a Hilbert transform (MATLAB function: *hilbert*) and subsequently taking the absolute value of the Hilbert-transformed signal (see Fig. 3.3). Finally, the correlation between both power envelopes was calculated, resulting in a single AEC value that describes the functional connectivity strength between two brain regions (see Fig. 3.4). This analysis was conducted for each epoch and frequency band separately, and the final AEC value was obtained by averaging the epoch-specific AEC values.

**Figure 3:**
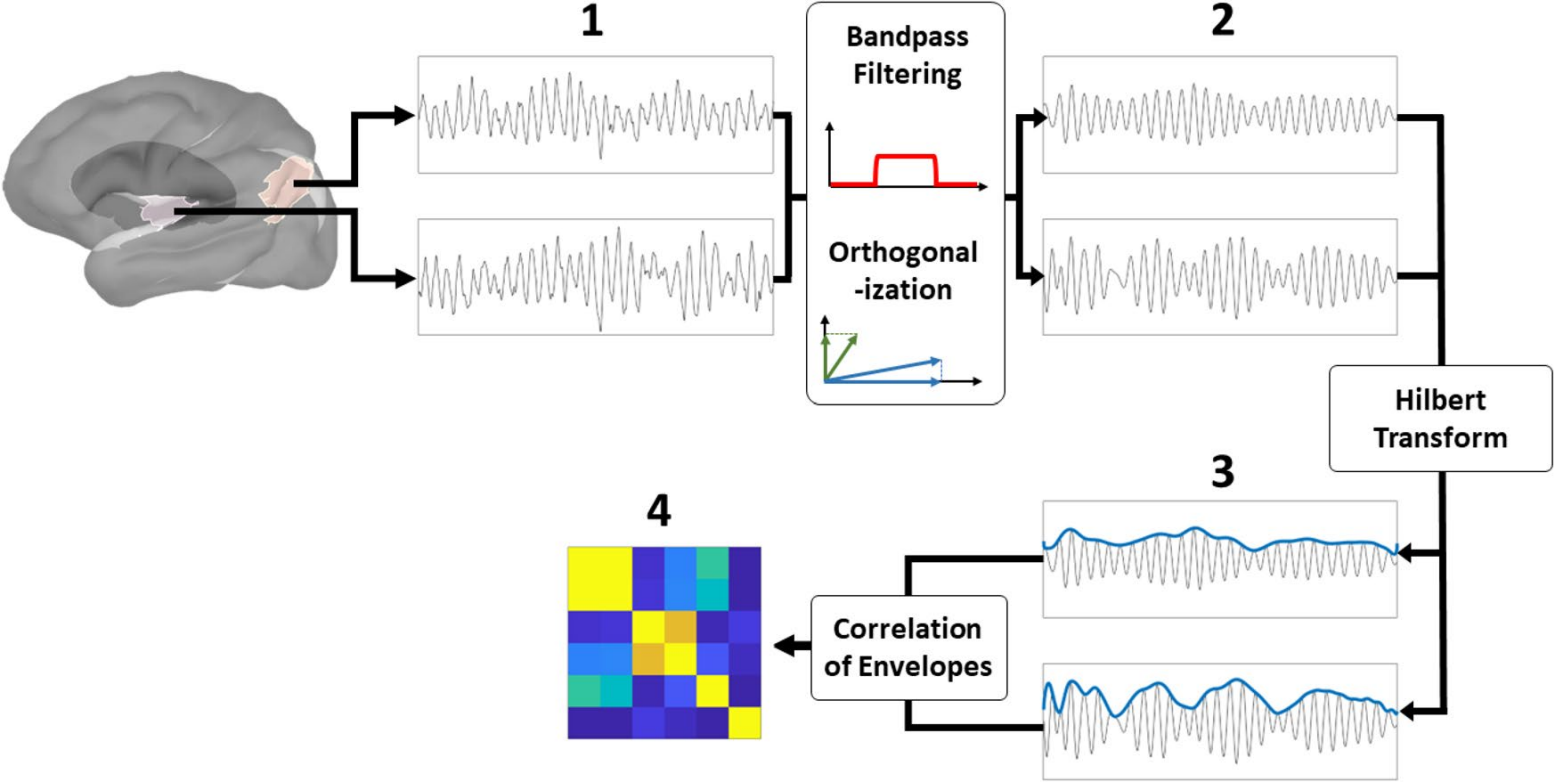
Visualization of the functional connectivity analysis. **(1)**: Selection of the timeseries of brain ROIs of interest. **(2)**: Preparing the timeseries by bandpass filtering (to only investigate the frequency band of interest) and orthogonalization (to eliminate spatial leakage due to the blurring of the minimum norm estimation). **(3)**: Computing the amplitude envelope of each timeseries using the Hilbert transform. **(4)**: Computing the correlation between the timeseries to obtain the AEC value. The orthogonalization step is performed twice, as are all subsequent steps for each set of orthogonalized timeseries.

Due to the large amount of possible functional connections which can be computed (for 10 brain regions and 3 frequency bands; 135 connections), initial results of the source power analysis were used to reduce the search space for the FC analysis. These initial results showed changes in activity within the alpha and beta frequency range and showed that the left and right precuneus seemed to be highly affected. Therefore only connections between the left or right precuneus and the other regions in the alpha and beta frequency range were considered. This led to 34 (17 connections, 2 frequency bands) possible connections. All source level functional connectivity analyses were performed using custom code in MATLAB. The scripts can be found on OSF (link: https://osf.io/tywxp).

### Statistical analysis

Statistical analysis was conducted using R (version 4.1.1.) and RStudio (version 2022.02.1.). All information regarding the statistical analysis can be found on OSF (the script: https://osf.io/u8zyv; information regarding used packages: https://osf.io/ekfcp; how to run the analysis: https://osf.io/vgz3x; how to convert EEG results to statistical tables: https://osf.io/389r4; the script of the Bayesian analysis : https://osf.io/76jev).

#### SAM analysis

For the SAM analysis (and all subsequent analyses), generalized linear mixed models (GLMMs) were employed (R package: *lme4*). The score on each scale (valence, arousal) was defined as a dependent variable, while the condition (control, negative) was used as a fixed effect and participant ID as a random effect (R formula: *Scale_score* ~ *Condition* + *(1|Participant_ID)*). Two models were trained: a linear mixed model (LMM) and a generalized linear mixed model (GLMM) using a gamma distribution and identity link. The models were compared using the Akaike Information Criterion (AIC), which assesses how much variance in the data is explained by the model (i.e., a lower AIC score indicates more variance is explained), and the model with the lowest AIC score was selected. The possible added value of participants’ sex was assessed by building a new model with sex included as a fixed effect (R formula: *Scale_Score* ~ *Condition* + *Sex* + *(1|Participant_ID)*) and comparing both models using an ANOVA test (type III). Given the preference for parsimonious models (i.e., models with fewer fixed or random effects are easier to interpret, and are less likely to overfit^79^), if the ANOVA showed that the model with sex as effect did not explain the variance in the data significantly better (i.e., the *p*-*value* of the ANOVA test is > 0.05) it was concluded that the sex of the participants did not contribute significantly and was therefore excluded from the model. See Kappen et al.^40^ for a detailed explanation.

#### ECG analysis

Analysis of the ECG-IBI data was similar to the SAM analysis. The twelve IBI-differences (IBI_-3_ to IBI_12_) were the dependent variable, condition (positive, negative) a fixed effect, and participant ID a random effect (R formula: *IBI-difference* ~ *Condition* + *(1|Participant_ID)*). The possible added value of sex was assessed identically as the SAM analysis (see section "SAM analysis"). Contrary to the SAM analysis, however, only a linear model was computed due to the presence of negative values for the dependent variable, which is incompatible with gamma distributions. See Kappen et al., (2022) for further explanations.

#### EEG power analysis

Before the statistical analysis of the EEG measures was performed, participants who did not have at least 15 epochs for both conditions (i.e., the neutral and negative feedback condition) were not included in the analysis, which lead to the exclusion of three participants. A full overview of the epoch amount for each participant can be found on OSF (link: https://osf.io/49azx).

##### Frequentist statistical analysis

For both the sensor and source power analyses, a similar approach as the SAM and ECG analysis was employed. The power values of either the mean of the frontal electrodes or the individual brain regions were the dependent variable, while the condition was a fixed effect and the participant ID a random effect (R formula: *PowerValue* ~ *Condition* + *(1|Participant)*). Both an LMM and GLMM were trained, given the fixed range of power values ([0, 1]; 0 = no power; 1 = all power) and model selection was performed using AIC (see section "SAM analysis"). The possible added value of sex was assessed identically as in section "SAM analysis". Estimated marginal means (EMMs) were computed (using the *emmeans* function), and the *p*-value from the *Condition* contrast was obtained. To gain further insight into the results, standardized effect sizes (SES, similar to Cohen’s D but adapted for non-normal disributions) and corresponding confidence intervals (CI) were computed (*eff_size* function).

##### Bayesian statistical analysis

For the sensor power analyses, a subsequent Bayesian analysis was conducted, as we hypothesized the absence of a significant difference between conditions. Firstly, two GLMMs with gamma distributions were trained for the power values of each frequency band separately. The first model was identical to the model described in the previous section, with the power value as dependent variable, the condition and participant sex as fixed effect and the participant ID as random effect (R formula: *PowerValue* ~ *Condition* + *Sex* + *(1|Participant)*). The second model (the null model) was trained without the condition as fixed effect (R formula: *PowerValue* ~ *Sex* + *(1|Participant)*). The Bayes factor (BF01) comparing both models was computed using the formula by Wagenmakers, which employs the Bayes Information Criterion (BIC) and does not require the definition of prior distributions^75^. BF01 compares the likelihood of the data under the null model, which did not include *condition* as a fixed effect (H_0_) with the likelihood of the data under the alternative model, which includes *condition* as fixed effect (H_A_). Larger BF01 values suggest the presence of evidence that favors the null hypothesis (H_0_) and the obtained BF01 factors were interpreted according to Jeffreys^76^.

#### EEG functional connectivity analysis

For FC values, statistical analyses were conducted similarly to section “EEG power analysis”. The main difference is that, since AEC has a range of [-1, 1], gamma GLMMs are not always possible given the inability of gamma GLMMs to work with non-positive values. Therefore, LMMs are always trained, and if possible (i.e., the currently considered functional connection does not have negative values for any participant) gamma GLMMs were trained. The further analysis was conducted as described in section “EEG power analysis”.

#### Multiple comparison correction

To correct for the multiple tests that were conducted (67; 3 (sensor level power), 30 (10 × 3 source level power), 34 (17 × 2 source level FC)), all obtained p-values were corrected for multiple comparisons using the false discovery rate correction method (FDR^77^). These FDR-corrected *p*-values are reported in the result section.

### Ethical approval

Ethical approval for the current study was obtained from the Medical Ethical committee of the Ghent University Hospital (registration number: B670201940636). 

### Consent to participate

All participants signed an informed consent form before participating to the study.

## Limitations

Some limitations in the current study can be noted. Firstly, the possibility of an order effect due to the positioning of the conditions is present. This positioning was chosen since counterbalancing would only have been possible when the resting time between conditions would be multiple hours (or days), given the long recovery phase of stressors^78^. However, as our results are in line with previous neuroimaging research (both fMRI and EEG), we believe that if present, order effects will have a minimal influence on our results. Secondly, the psychosocial stressor that was employed is subtle, which likely reduces the effect sizes. This makes it difficult to generalize our findings to all psychosocial stressors. Thirdly, the feedback screen consisted of multiple parts, so it might be possible that some confounding effect are introduced. Fourthly, the amount of EEG electrodes is relatively low for EEG source imaging. Related to this, a template MRI image is also used, which does not perfectly match the brain structure of the participants. The sizes of the chosen source level brain regions however are quite large, which makes the misattribution of electrical activity to regions not likely. Finally, it should be mentioned that aside from the brain regions investigated in this study, other regions, mainly subcortical regions not accessible by EEG due to their deep location within the brain, are also consistently reported in previous studies. It is likely that, to better understand the reaction of the brain to psychosocial stressors, these regions should also be considered.

## Data Availability

All data, except for the raw EEG data, is available on OSF (link: https://osf.io/5qew6/).

## Code availability

Code is available on OSF (link: https://osf.io/5qew6) and Github (link: https://github.com/dx2r/PhD_EEG_Pipeline).
